# Acute Pancreatitis and Rhabdomyolysis with Acute Kidney Injury following Multiple Wasp Stings

**DOI:** 10.1155/2017/8596981

**Published:** 2017-06-19

**Authors:** Seo Hee Yang, Yeon Han Song, Tae Hoon Kim, Su Bin Kim, Sang Youb Han, Han-Seong Kim, Se Won Oh

**Affiliations:** ^1^Department of Internal Medicine, Inje University College of Medicine, Goyang, Republic of Korea; ^2^Department of Pathology, Inje University College of Medicine, Goyang, Republic of Korea

## Abstract

Multiple wasp stings can induce multiple organ dysfunction by toxic reactions. However, acute pancreatitis is a rare manifestation in wasp sting injury. A 74-year-old woman visited the emergency department by anaphylactic shock because of multiple wasp stings. Acute kidney injury, rhabdomyolysis, hepatotoxicity, and coagulopathy were developed next day. Serum amylase and lipase were elevated and an abdominal computed tomography revealed an acute pancreatitis. Urine output was recovered after 16 days of oliguria (below 500 ml/day). Her kidney, liver, and pancreas injury gradually improved after sessions of renal replacement therapy.

## 1. Introduction

Wasp stings are common worldwide, and the responses to wasp stings are diverse, ranging from mild local reaction to severe systemic reactions [1, 2]. It is estimated that systemic reactions develop in 0.15–3.3% of wasp sting injuries that occur in the general population [3]. Mass envenomation can evoke severe systemic manifestations such as acute kidney injury, hepatotoxicity, coagulopathy, respiratory distress, and shock [4]. However, reports of acute pancreatitis that develops after wasp stings are rare. We report a case with multiple organ failure including acute kidney injury (AKI), rhabdomyolysis, liver injury, coagulopathy, and acute pancreatitis, following multiple wasp stings.

## 2. Case Report

A 74-year-old woman visited the emergency department in our institute after multiple wasp stings. She complained of dizziness and whole body pain. More than 40 stings marks were found across her head, neck, both upper and lower extremities, buttocks, and back. On admission, blood pressure was 60/49 mmHg and pulse rate was 120 per minute. She was given intravenous fluids, hydrocortisone, and antihistamines. Her vital signs and symptoms recovered, and she was discharged. The next day, she returned to the hospital complaining of dyspnea, nausea, general ache, edema, and decreased amount of urine. She has a history of diabetes mellitus and hypertension for 20 years. She had chronic kidney disease stage III, and her estimated glomerular filtration rate (eGFR) has been measured at 55.4 ml/min/1.73 m^2^ four months before the wasp stings. She had undergone surgery and chemotherapy for colon cancer nine years ago. She was taking medications including telmisartan 40 mg, pravastatin 10 mg, glimepiride 2 mg, metformin 500 mg, and acetylsalicylic acid 100 mg.

Her mentality was drowsy, and blood pressure was 158/83 mmHg, pulse rate 118/min, body temperature 36.2°C, and respiratory rate 20/min. There was mild tenderness in the epigastric area without rebound tenderness. Urine color was dark brown, and the hourly urine output was 10–20 cc. The initial laboratory results were serum hemoglobin 11.7 g/dL, white blood cell count 18,200 cells per microliter, platelets 213,000/uL, blood urea nitrogen 66 mg/dL, creatinine 3.97 mg/dL, eGFR 10.6 ml/min/1.73 m^2^, sodium 130 meq/L, potassium 8.0 meq/L, ionized calcium 1.01 mmol/L, phosphorous 12.5 mg/dL, serum bilirubin 4.7 mg/dL, aspartate aminotransferase 10380 IU/L, alanine aminotransferase 3302 IU/L, lactate dehydrogenase 8356 IU/L (reference: 140–271 IU/L), creatinine kinase (CK) 98,862 IU/L (reference: <171 IU/L), PT 18 sec (reference: 11.8–14.3 sec), PTT 121.3 sec (reference: 29–44 sec), D-dimer 4499.69 ng/mL (reference: <500 ng/mL), FDP 5~20 ug/mL (reference: <5 ug/mL), fibrinogen 441 mg/dL (reference: 180–400 mg/dL), amylase 205 IU/L (reference: 22–85 IU/L), and lipase 622 IU/L (reference: 21–67 U/L). Arterial blood gas analysis results were pH 7.11, pCO2 26 mmHg, HCO3 9.5 mmol/L, and oxygen saturation 93%. Urine analysis revealed a 1.025 specific gravity, protein 3+, occult blood 4+, RBC 5–9/HPF, and WBC 1–4/HPF. Viral markers for hepatitis B, hepatitis C, and HIV were negative. Chest X-ray did not show pulmonary edema or abnormal infiltration. Electrocardiography revealed sinus tachycardia, absent P wave, and tall T wave.

**Figure 1:**
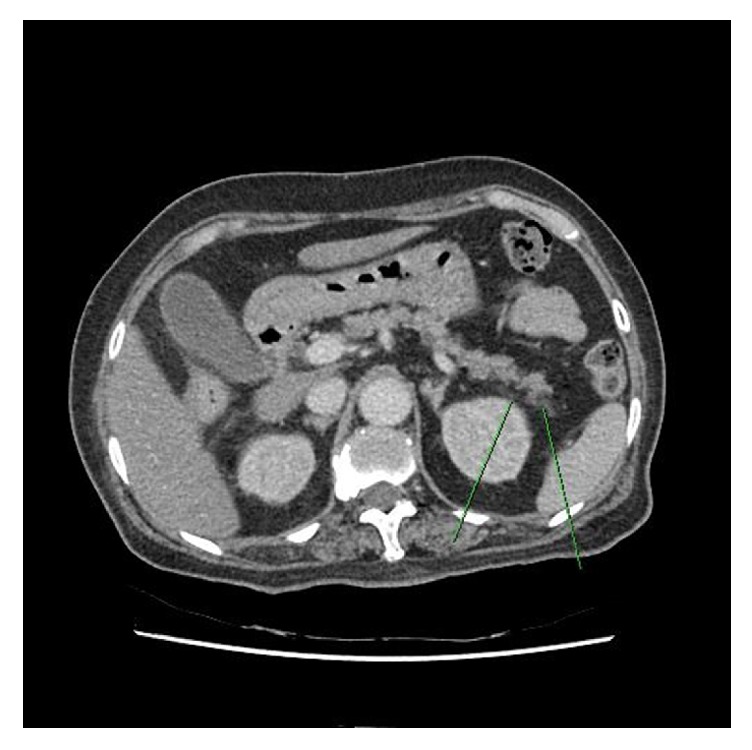
Abdominal computer tomography (CT) scan shows acute pancreatitis. Contrast enhanced abdominal CT scan shows fat infiltration in the tail portion of the pancreas parenchyma, suggesting acute pancreatitis grade C.

**Figure 2:**
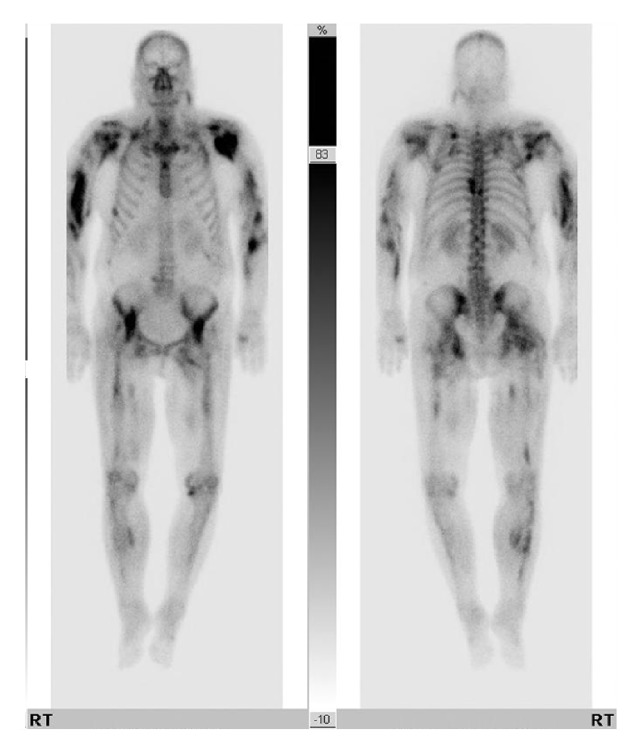
Whole body bone scintigraphy shows radioiodine uptake in multiple locations. Whole body bone scintigraphy shows radioiodine uptake in the head, shoulder, hip, and both upper and lower extremities, suggesting rhabdomyolysis.

**Figure 3:**
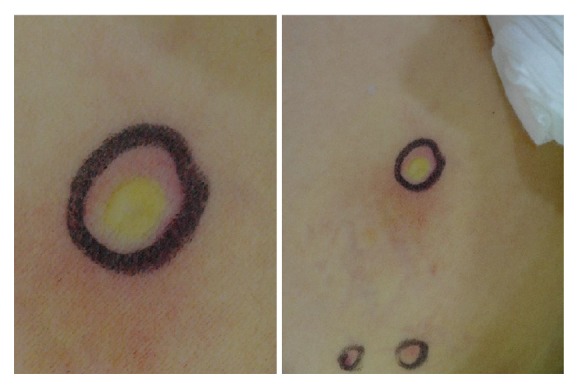
Erythema and blister formation on the skin.

Abdominal computed tomography showed peripancreatic fat infiltration in the tail portion, suggesting acute pancreatitis grade C (Figure 1). Whole body bone scintigraphy showed multiple radioiodine uptake in the head, shoulder, hip, and both upper and lower extremities (Figure 2). Approximately 1–3-cm sized blisters were formed at the sites of the wasp stings and lasted for one week (Figure 3). The skin biopsy showed subdermal bullae with eosinophilic infiltration.

Continuous renal replacement therapy (CRRT) was initiated 30 hours after the wasp stings. Serum CK level and liver function test were significantly improved during maintenance of CRRT for three days. Serum amylase and lipase level were decreased to less than three times the upper reference limit six days after the wasp stings. However, AKI was persistent and peak serum creatinine level was 8.58 mg/dL on the 13th day after admission. We underwent 11 sessions of conventional hemodialysis until renal function was restored. Ten months after the wasp stings, her serum creatinine level had improved to 1.6 mg/dL (eGFR 30 ml/min/1.73 m^2^).

## 3. Discussion

Wasp stings occur worldwide. Almost patients suffered from a few stings with a variable degree of allergic reactions [1–3]. However, multiple wasp envenomation has been reported to cause life-threatening multiple organ injuries [4, 5]. Our patient showed a variety of organ injuries such as anaphylactic shock, rhabdomyolysis, liver injury, coagulation disorder, acute kidney injury, and acute pancreatitis due to numerous wasp stings.

The clinical symptoms resulting from wasp sting injuries can be classified into allergic and toxic reactions. Allergic reactions may involve dermal symptoms (erythema, pruritus, urticaria, and angioedema), respiratory distress (upper airway edema and bronchial constriction), and cardiovascular collapse (anaphylactic shock and cardiac arrest) [2, 5, 6]. Localized allergic reactions are usually self-limiting, lasting for 2-3 days. Systemic allergic reactions are known as an immediate-type hypersensitivity mediated by venom-specific IgE after prior sensitization. Serious systemic allergic reactions can occur within minutes of the sting injury [2, 6]. However, a recent report showed that deaths were more frequently caused by toxicity to organs, rather than anaphylactic shock [5]. In patients that are hospitalized with wasp stings, the main clinical manifestations were toxic reactions such as rhabdomyolysis, hemolysis, hepatotoxicity, coagulation disorders, and AKI [5, 7–9]. In the presented patient, anaphylactic shock was observed a few hours after wasp stings, at admission, and it was improved by hydrocortisone and antihistamine. However, she complained of generalized edema and oliguria the next day, and AKI was diagnosed. The onset of AKI following wasp stings is known to be caused by hypotension resulting from anaphylactic or hypovolemic shock, acute tubular necrosis, acute interstitial nephritis, and pigment nephropathy (resulting from rhabdomyolysis and hemolysis) [7–9]. In this patient, severe rhabdomyolysis was diagnosed by elevated levels of muscle enzymes, and radioiodine accumulation in parts of the body, which was detected by bone scintigraphy. Severe rhabdomyolysis was considered the cause of AKI. In addition to AKI, hepatotoxicity, coagulopathy, and acute pancreatitis were noted.

Acute pancreatitis due to wasp sting had been rarely reported [10–12]. Phospholipase A2 (PLA2) is a major component of wasp venom [2, 8, 10]. PLA2 catalyzes the hydrolysis of 2-acyl bonds in phospholipids and decomposes the phospholipidic part of cell membranes [13]. PLA2 has a toxic effect on striated muscle, lyses red blood cells, increases capillary permeability, destroys mast cells, and causes abnormal coagulation. These actions of PLA2 lead to the development of rhabdomyolysis [8, 10–12]. In addition, the PLA2 component of the venom may play a role in the development of acute pancreatitis following wasp stings. PLA2 is mainly released to the blood from neutrophilic granulocytes, macrophages, and platelets in the pancreatic acini when acute pancreatitis occurs [13]. In acute pancreatitis, the level of serum PLA2 correlates with disease severity and prognosis [14]. PLA2 causes massive leakage of lysosomal enzymes out of cells by attacking the stability of cell membranes and provoking visceral injury to the pancreas and other tissues [13]. In reports of patients with acute pancreatitis following wasp stings, the prognoses predicted fatal outcomes [10, 11]. A previous report showed that renal replacement therapy improved the prognosis of patients that have multiple wasp sting injuries [5, 15]. CCRT can remove venom toxins and inflammatory mediators, as well as the myoglobin released into the bloodstream because of rhabdomyolysis [15]. Although our patient had risk factors for poor prognosis such as underlying chronic kidney disease and multiple wasp sting injuries, adequate renal replacement therapy greatly improved prognosis [5].

For patients who experience multiple wasp stings, early detection and management of multiple organ injury is critical. The patient with AKI, rhabdomyolysis, and acute pancreatitis was adequately treated with CRRT.
